# Indicators for maternal near miss: an observational study, India

**DOI:** 10.2471/BLT.21.287737

**Published:** 2022-06-02

**Authors:** Divya Mecheril Balachandran, Dhamotharan Karuppusamy, Dilip Kumar Maurya, Sitanshu Sekhar Kar, Anish Keepanasseril

**Affiliations:** a Department of Obstetrics & Gynaecology, Jawaharlal Institute of Postgraduate Medical Education & Research, Dhanvantri Nagar, Puducherry, 605006, India.; b Department of Preventive and Social Medicine, Jawaharlal Institute of Postgraduate Medical Education & Research, Puducherry, India.

## Abstract

**Objective:**

To compare the incidence of maternal near miss using the World Health Organization (WHO) near-miss tool and six other criteria sets, including criteria designed for low-resource settings or specifically for India.

**Methods:**

In a cohort study we used WHO severity indicators to identify women with potentially life-threatening conditions during pregnancy or childbirth admitted to a referral hospital in Puducherry, India, from May 2018 to April 2021. We analysed sociodemographic, clinical and laboratory data for each woman and calculated the incidence of maternal near miss and other process indicators for each set of criteria.

**Findings:**

We analysed data on 37 590 live births; 1833 (4.9%) women were identified with potentially life-threatening conditions, 380 women had severe maternal outcomes and 57 died. Applying the different sets of criteria to the same data, we found the incidence of maternal near miss ranged from 7.6 to 15.6 per 1000 live births. Only the Global Network criteria (which exclude laboratory data that may not be available in low-resource settings) and the WHO criteria could identify all women who died. Applying the criterion of any number of units of blood transfusion increased the overall number of women identified with near miss.

**Conclusion:**

The WHO and Global Network criteria may be used to detect maternal near miss in low-resource settings. Future studies could assess the usefulness of blood transfusion as an indicator for maternal near miss, especially in low- to middle-income countries where the indicator may not reflect severe maternal morbidity if the number of units received is not specified.

## Résumé

**Objectif:**

Établir une comparaison de l'incidence des décès maternels évités de justesse en utilisant l'outil mis au point par l'Organisation mondiale de la Santé (OMS) ainsi que six autres ensembles de critères, notamment ceux conçus pour les milieux à faibles ressources ou, plus spécifiquement, pour l'Inde.

**Méthodes:**

Dans le cadre d'une étude de cohorte, nous avons appliqué les indicateurs de gravité de l'OMS afin d'identifier les femmes dont l'état de santé pouvait s'avérer potentiellement mortel durant la grossesse ou l'accouchement, admises dans un hôpital de référence à Pondichéry, en Inde, entre mai 2018 et avril 2021. Nous avons examiné les données sociodémographiques, cliniques et de laboratoire relatives à chacune de ces femmes. Nous avons ensuite calculé l'incidence des décès maternels évités de justesse et d'autres indicateurs de processus pour chaque ensemble de critères.

**Résultats:**

Notre analyse portait sur 37 590 naissances vivantes; 1833 femmes (4,9%) ont été considérées comme présentant des risques élevés, 380 femmes ont connu de graves problèmes de santé, et 57 sont décédées. En étudiant les mêmes données à travers le prisme des différents ensembles de critères, nous avons découvert que l'incidence des décès maternels évités de justesse était comprise entre 7,6 et 15,6 pour 1000 naissances vivantes. Seuls les critères du Réseau mondial (qui ne reprend pas les données de laboratoire non disponibles dans les milieux à faibles ressources) et de l'OMS ont permis d'identifier toutes les femmes n'ayant pas survécu. Appliquer le critère tenant compte du nombre d'unités de sang transfusé, quel qu'il soit, a fait augmenter le nombre total de femmes considérées comme ayant échappé de justesse à un décès.

**Conclusion:**

Les critères de l'OMS et du Réseau mondial peuvent servir à détecter les décès maternels évités de justesse dans les milieux à faibles ressources. Les futures études sur le sujet pourraient évaluer l'utilité de la transfusion sanguine en tant qu'indicateur, en particulier dans les pays à revenu faible et intermédiaire où cet indicateur pourrait ne pas refléter la morbidité maternelle grave si le nombre d'unités transfusées n'est pas précisé.

## Resumen

**Objetivo:**

Comparar la tasa de incidencia de la morbilidad materna extrema mediante la herramienta de morbilidad extrema de la Organización Mundial de la Salud (OMS) y otros seis conjuntos de criterios, incluidos los criterios elaborados para entornos de bajos recursos o específicos para la India.

**Métodos:**

A través de un estudio de cohortes, se utilizaron los indicadores de gravedad de la OMS para identificar a las mujeres con afecciones potencialmente mortales durante el embarazo o el parto ingresadas en un hospital de referencia en Puducherry, India, desde mayo de 2018 hasta abril de 2021. Se analizaron los datos sociodemográficos, clínicos y de laboratorio de cada mujer y se calculó la tasa de incidencia de la morbilidad materna extrema y otros indicadores de proceso para cada conjunto de criterios.

**Resultados:**

Se analizaron los datos de 37 590 nacidos vivos; se identificaron 1833 (4,9 %) mujeres con afecciones potencialmente mortales, 380 mujeres presentaron desenlaces maternos graves y 57 murieron. Al aplicar los diferentes conjuntos de criterios a los mismos datos, se observó que la tasa de incidencia de la morbilidad materna extrema oscilaba entre 7,6 y 15,6 por cada 1000 nacidos vivos. Solo los criterios de la Red Mundial (que excluyen los datos de laboratorio que podrían no estar disponibles en entornos de bajos recursos) y los criterios de la OMS pudieron identificar a todas las mujeres que murieron. La aplicación del criterio de cualquier número de unidades de transfusión de sangre aumentó el número total de mujeres identificadas con morbilidad extrema.

**Conclusión:**

Los criterios de la OMS y de la Red Mundial se podrían utilizar para detectar la morbilidad materna extrema en entornos de bajos recursos. En futuros estudios, se podría evaluar la utilidad de la transfusión de sangre como indicador para detectar la morbilidad materna extrema, en especial en los países de ingresos bajos y medios, donde el indicador podría no reflejar la morbilidad materna grave si no se especifica el número de unidades de transfusión recibidas.

## ملخص

الغرض مقارنة حدوث الحالات وشيكة الوفاة للأمهات باستخدام أداة "الحالات وشيكة الوفاة" فضلاً عن 6 مجموعات أخرى من المعايير التابعة لمنظمة الصحة العالمية (WHO)، بما في ذلك المعايير المصممة للظروف منخفضة الموارد، أو بخاصة للهند.

الطريقة في دراسة إترابية، قمنا باستخدام مؤشرات الشدة الخاصة بمنظمة الصحة العالمية لتحديد النساء اللواتي يعانين من ظروف قد تهدد الحياة أثناء الحمل أو الولادة، تم إدخالهن إلى مستشفى إحالة في بودوتشيري بالهند، من مايو/أيار 2018 إلى أبريل/نيسان 2021. قمنا بتحليل البيانات الاجتماعية السكانية، والسريرية والمختبرية لكل امرأة، وحساب حدوث الحالات وشيكة الوفاة للأمهات، ومؤشرات العملية الأخرى لكل مجموعة من المعايير.

النتائج قمنا بتحليل بيانات 37590 من المواليد الأحياء؛ وتم تحديد عدد 1833 من السيدات (بنسبة 4.9%) مصابات بأعراض قد تهدد الحياة، و380 من السيدات عانين من نتائج وخيمة بعد الولادة وتوفيت منهن 57 سيدة. بتطبيق مجموعات مختلفة من المعايير على نفس البيانات، وجدنا أن معدل حدوث الحالات وشيكة الوفاة للأمهات يتراوح من 7.6 إلى 15.6 لكل 1000 من المواليد الأحياء. يمكن فقط لمعايير الشبكة العالمية (التي تستبعد البيانات المختبرية التي قد لا تكون متوفرة في ظروف منخفضة الموارد) ومعايير منظمة الصحة العالمية، تحديد جميع النساء اللاتي توفين. أدى تطبيق معيار أي عدد من وحدات نقل الدم إلى زيادة العدد الإجمالي للسيدات اللواتي يعانين من الوفاة الوشيكة.

الاستنتاج يمكن استخدام معايير منظمة الصحة العالمية والشبكة العالمية للكشف عن الحالات وشيكة الوفاة للأمهات في ظروف منخفضة الموارد. يمكن للدراسات المستقبلية تقييم مدى فائدة نقل الدم كمؤشر لحالات الوفاة الوشيكة للأمهات، وبخاصة في الدول ذات الدخل المنخفض والدخل المتوسط، حيث قد لا يعكس المؤشر معدلات مرتفعة لإصابة الأمهات بالأمراض، إذا لم يتم تحديد عدد الوحدات التي تم تلقيها.

## 摘要

目的 使用世卫组织 (WHO) 的危重症评估工具和其他六组标准（包括为资源匮乏地区或专为印度制定的标准），比较孕产妇危重症的发生率。

方法 在一项队列研究中，我们使用世卫组织严重程度指标来识别 2018 年 5 月至 2021 年 4 月在印度普杜切里转诊医院收治的怀孕或分娩期间可能危及生命的妇女。我们分析了每位妇女的社会人口学、临床和实验室数据，并计算了每组标准的孕产妇危重症发生率以及其他过程指标。

结果 我们分析了 37,590 例活产婴儿的数据；识别了 1,833 名妇女可能患有危及生命的疾病，380 名妇女出现严重的孕产妇结局，57 名妇女死亡。将不同组的标准应用于相同的数据，我们发现每 1,000 个活产婴儿中孕产妇危重症的发生率为 7.6 至 15.6。只有全球网络标准（排除在资源匮乏地区可能无法获得的实验室数据）和世卫组织标准才能识别所有死亡妇女。采用任意数量的输血单位这样的标准增加了被识别为危重症孕产妇的总人数。

结论 世卫组织和全球网络标准可用于识别资源匮乏地区的危重症孕产妇。未来的研究可以评估输血作为孕产妇危重症指标的有效性，特别是在中低收入国家，如果未说明接受的输血单位具体数量，则该指标可能无法反映孕产妇的危重症发病率。

## Резюме

**Цель:**

Сравнить частоту осложнений при родах, представляющих угрозу для жизни, с использованием соответствующего инструмента Всемирной организации здравоохранения (ВОЗ) и шести других наборов критериев, включая критерии, разработанные для условий с ограниченными ресурсами или специально для Индии.

**Методы:**

В когортном исследовании авторы использовали показатели степени тяжести, разработанные ВОЗ, для выявления женщин с потенциально опасными для жизни состояниями во время беременности или родов, поступивших в лечебно-диагностические центры в г. Пондичерри, Индия, с мая 2018 г. по апрель 2021 г. Авторы проанализировали социально-демографические, клинические и лабораторные данные для каждой женщины и рассчитали частоту осложнений при родах, представляющих угрозу для жизни, а также другие показатели процесса для каждого набора критериев.

**Результаты:**

Авторы проанализировали данные о 37 590 живорожденных. У 1833 женщин (4,9%) были выявлены потенциально опасные для жизни состояния, у 380 женщин были тяжелые исходы родов, а 57 женщин умерли. Применяя различные наборы критериев к одним и тем же данным, авторы обнаружили, что частота осложнений при родах, представляющих угрозу для жизни, колеблется от 7,6 до 15,6 на 1000 живорожденных. Только критерии Глобальной сети (исключающие лабораторные данные, которые могут быть недоступны в условиях ограниченных ресурсов) и критерии ВОЗ могут выявить всех умерших женщин. Применение критерия любого количества единиц переливания крови увеличивало общее число женщин, у которых выявлено осложнение при родах, представляющее угрозу для жизни.

**Вывод:**

Критерии ВОЗ и Глобальной сети могут быть использованы для выявления осложнений при родах, представляющих угрозу для жизни, в условиях ограниченных ресурсов. В будущих исследованиях можно было бы оценить полезность переливания крови как показателя осложнений при родах, представляющих угрозу для жизни, особенно в странах с низким и средним уровнем дохода, где этот показатель может не отражать уровень серьезных осложнений у матерей, если количество полученных единиц не указано.

## Introduction

Maternal mortality refers to the “death of a woman whilst pregnant or within 42 days of delivery or termination of pregnancy, from any cause related to, or aggravated by pregnancy or its management, but excluding deaths from incidental or accidental causes.”^1^ While many women die following a life-threatening event, many more women survive with morbidity and long-term sequelae. Survival after a life-threatening event is referred to as maternal near miss, defined as “a woman who nearly died but survived a complication that occurred during pregnancy, childbirth or within 42 days of termination of pregnancy.”^2^ Monitoring these near-miss events will provide insight into the quality of obstetric care offered in a facility including the strength and weakness of the referral system and availability of clinical interventions, which could suggest improvements to reduce severe maternal complications.^2^^,^^3^

An ideal system or set of criteria to identify maternal near miss should (i) be easy to implement (with a minimal number of severity indicators); (ii) not miss those who may succumb to the disease process; and (iii) be possible to use in all settings, allowing comparison of the incidence of near-miss events similar to the maternal mortality ratio which is currently used. High-income countries with electronic health records use the International Classification of Disease (ICD) codes to diagnose near-miss events, whereas lower income countries rely more on clinical and management criteria for the diagnosis.^4^^–^^7^

The World Health Organization (WHO) first defined surveillance recommendations for monitoring near miss in 2009^2^^,^^8^ and published the *WHO near-miss approach for maternal health* in 2011.^9^ Even after a decade of using the WHO criteria, differences in the available data limits the use of near miss as an indicator for maternal health care in comparing across different countries and regions within the same country. Several authors have made modifications to the criteria, citing resource constraints that prevent use of the WHO approach. The modifications range from adding specific clinical conditions or modifying the indicators, to approaches in which a combination of individual indicators (one each from clinical, investigation and management) are used to define near miss.^5^^,^^10^^–^^14^ The Global Network Near-Miss Maternal Mortality System is one such approach, which omitted the laboratory criteria for maternal near miss, as centres with limited resources lack these facilities.^15^ For this study in India, we aimed to compare the incidence of maternal near-miss events in women with potentially life-threatening conditions during pregnancy or childbirth, calculated using different sets of criteria. We used the WHO proposed criteria and six others, including the country-specific consensus criteria developed by the Indian national technical group in 2014.^16^

## Methods

### Study design and setting

We based the study on data collected as part of a primary study assessing the incidence of near-miss events and the impact of the event on maternal health at 12 months, among a cohort of women with potentially life-threatening conditions admitted to hospital from May 2018 to April 2021. The setting was the Women and Children’s Hospital of Jawaharlal Institute of Postgraduate Medical Education and Research, Puducherry, India. Situated in the south-eastern coastal region of India, the hospital caters primarily for a rural population and manages 17 000–18 000 deliveries annually. The hospital provides tertiary care to women with high-risk pregnancies referred from the Union Territory of Puducherry and the neighbouring districts of Tamil Nadu.

### Study population

Women older than 18 years admitted with a potentially life-threatening condition, as defined by WHO, were included in the study.^2^^,^^8^ The criteria include: (i) haemorrhagic disorders such as placental abruption, placenta praevia, postpartum haemorrhage, ectopic pregnancy and ruptured uterus; (ii) hypertensive disorders such as severe pre-eclampsia, eclampsia, hypertensive urgencies and HELLP (haemolysis, elevated liver enzymes, low platelet count) syndrome; (iii) other systemic disorders such as pulmonary oedema, seizures, sepsis, shock, thrombocytopenia (platelet count < 100 × 10^9^/L) and thyroid crisis; and (iv) management indicators such as blood transfusions, central venous access, and hysterectomy or surgical intervention.^17^ The women were recruited from the intensive care units, eclampsia room and high dependency unit.

### Data collection

After taking informed written consent, the research staff collected sociodemographic information on each woman with potentially life-threatening conditions, including age, level of education, obstetric and medical history, the care received in the hospital until discharge, and neonatal outcomes. 

### Criteria sets

In addition to the WHO near-miss tool, we studied six other commonly used sets of criteria for determining maternal near miss which we identified in a literature search (Table 1).^7^^,^^15^^,^^16^^,^^18^^,^^19^^,^^22^ The Mantel^18^ and Waterstone^19^ criteria use organ dysfunction and clinical diagnosis for predicting near miss and we included them in this study for their ease of use in low- and middle-income settings. The Global Network criteria^15^ are a modification of the WHO criteria, which exclude laboratory criteria for the diagnosis of near miss to reflect the situation in low- and middle-income countries where the availability of laboratory services is limited. In contrast, the United States Centers for Disease Control and Prevention (CDC) criteria^20^^,^^22^ and the maternal morbidity outcomes indicators proposed by Roberts et al.^7^ are based on ICD diagnosis and procedures codes that are primarily used in high-income settings which have electronic health records or a population database.^7^^,^^15^^,^^20^^,^^22^ Finally, we applied the Indian consensus criteria that are recommended by the Indian government and are being used in some centres.^16^ The Indian national criteria are more complex than the other approaches to identifying maternal near miss and are detailed elsewhere.^23^

**Table 1:** Criteria sets for the diagnosis of maternal near miss used in the study of maternal near miss, Puducherry, India

Variable	Mantel et al., 1998^18^	Waterstone et al., 2001^19^	Roberts et al., 2008^7^	WHO criteria, 2011^9^	Chou et al., 2016^15^ (Global Network criteria)	CDC criteria, 2017^20^
**Basis of criteria**	• Clinical (on organ dysfunction) and management	• Clinical diagnosis	• ICD-code (diagnosis or procedures)	• Clinical laboratory and management	• Clinical or management	• ICD-code
**Usefulness in low- and middle- income countries **	• Possible to use, as clinical	• Possible to use, as clinical	• Difficult to use, as based on electronic data or records	• Suitable to use, although laboratory services may not be uniformly available	• Possible and suitable to use, as only clinically and management based	• Difficult to use, as based on electronic data or records
**Indicators**						
Cardiovascular	• Pulmonary oedema • Cardiac arrest	• Severe pre-eclampsia	• Shock • Cardiac failure • Cardiac arrest or infarction	• Shock • Cardiac arrest • Use of continuous vasoactive drugs • Cardiopulmonary resuscitation • pH < 7.1 • Lactate > 5 mmol/L	• Shock • Cardiac arrest • Use of continuous vasoactive drugs • Cardiopulmonary resuscitation	• Acute myocardial infarction or aneurysm • Cardiac arrest or ventricular fibrillation • Conversion of cardiac rhythm • Pulmonary oedema or acute heart failure • Shock
Respiratory	• Intubation and ventilation for ≥ 60 minutes unrelated to general anaesthesia • Oxygen saturation < 90% for ≥ 60 minutes, PaO_2_/FiO_2_ ratio ≤ 300 mmHg	NA	• Obstetric embolism • Acute severe asthma • Assisted ventilation including tracheostomy	• Acute cyanosis • Gasping • Respiratory rate > 40 or < 6 breaths per minute • Intubation and ventilation not related to anaesthesia • Oxygen saturation < 90% for ≥ 60 minutes • PaO_2_/FiO_2_ ratio < 200 mmHg	• Acute cyanosis • Gasping • Respiratory rate > 40 or < 6 breaths per minute • Intubation and ventilation not related to anaesthesia	• Adult respiratory distress syndrome • Amniotic fluid embolism • Air embolism or thrombotic embolism • Temporary tracheostomy • Ventilation
Renal	• Oliguria (≤ 400 mL in 24 hours), not responding to rehydration or diuresis • Urea > 15 mmol/L • Creatinine > 400 mmol/L	NA	• Acute renal failure • Dialysis	• Oliguria non-responsive to fluids • Dialysis for acute renal failure • Creatinine ≥ 300 mmol/L or ≥ 3.5 mg/dL	• Non-responsive to fluids • Dialyses for acute renal failure	• Acute renal failure
Haematological	• Acute thrombocytopenia requiring platelet transfusion • Transfusion of ≥ 5 units of blood or packed red cells	• Severe bleeding	• Any transfusion of blood or coagulation factors • Disseminated intravascular coagulation • Sickle cell anaemia with crisis	• Failure to form clots • Transfusion of ≥ 5 units of blood or packed red blood cells • Acute severe thrombocytopenia (platelet count ≤ 50 000/mm^3^)	• Failure to form clots • Blood transfusion (any volume)	• Transfusion of blood products • Disseminated intravascular coagulation • Sickle cell disease with crisis
Hepatic	• Jaundice in the presence of pre-eclampsia	HELLP syndrome	NA	• Jaundice with pre-eclampsia • Bilirubin > 100 mmol/L or > 6.0 mg/dL	• Jaundice with pre-eclampsia • Eclampsia	NA
Neurological	• Coma, lasting for > 12 hours • Subarachnoid or intracerebral haemorrhage	• Eclampsia	• Cerebral oedema or coma • Status epilepticus^a^ • Cerebrovascular accident	• Loss of consciousness or coma (lasting > 12 hours) • Stroke • Status epilepticus or uncontrollable fits • Total paralysis	• Loss of consciousness • Stroke • Fits • Paralyses	• Eclampsia • Puerperal cerebrovascular disorders
Immunological	• Sepsis leading to intensive care admission	• Severe sepsis	NA	NA	NA	• Sepsis
Metabolic	• Diabetic ketoacidosis • Thyroid crisis	NA	NA	NA	NA	NA
Procedures	• Emergency hysterectomy for sepsis or any other reason	• Ruptured uterus	• Uterine rupture • Repair of rupture of inverted uterus • Reclosure of disrupted caesarean section wound • Evacuation of haematoma • Hysterectomy • Dilatation and curettage under general anaesthesia • Interventions to control bleeding • Repair of bladder or cystostomy • Repair of intestine	• Haemorrhage leading to hysterectomy	• Surgical procedure to stop bleeding	• Hysterectomy • Heart failure or arrest during procedure
Anaesthesia-related	• Severe hypotension (with spinal or epidural anaesthetic) • Failed tracheal intubation requiring anaesthetic reversal	NA	• Major anaesthesia complications	NA	NA	• Severe anaesthesia complications
Other	• Intensive care unit admission for any reason	NA	• Acute abdomen • Acute psychosis • Acute appendicitis	NA	NA	NA

CDC: United States Centers for Disease Control and Prevention; HELLP: haemolysis, elevated liver enzymes and low platelet count; ICD: International Statistical Classification of Diseases and Related Health Problems, 10th revision; NA: not applicable; WHO: World Health Organization.

^a^ Status epilepticus is defined as continuous, generalized, convulsive seizure lasting > 5 minutes, or two or more seizures during which the patient does not return to baseline consciousness.^21^

### Outcomes and analysis

First, we determined the number of women with severe maternal outcome (those who have a life-threatening event) and maternal near miss, using the 25 severity indicators proposed in the WHO near-miss tool.^2^ We present the baseline characteristics of the women, the various potentially life-threatening conditions and the indicators in the WHO criteria as frequencies and percentages. Case fatality of each potentially life-threatening condition is presented as a percentage. We calculated the variable by dividing the number of deaths following a potentially life-threatening condition with the total number of women with that particular condition.

To assess differences in the rates of recognition of severe morbidity and maternal near-miss events using the various sets of criteria, we calculated the number of maternal deaths per 100 000 live births and the near-miss indicators for each criteria set.^2^ We did not report diagnostic accuracy parameters to compare the methods using one criterion set as a reference standard. Instead, we compared the number of maternal near misses and the number of maternal deaths (and other maternal near-miss indicators) identified by each criteria set. This approach aims to reflect the burden that near misses will add (due to overdiagnosis) or reduce (missing those who ultimately die due to the complications).

We calculated the following indicators:^2^ (i) maternal near-miss ratio per 1000 live births, calculated from: [(number of maternal near misses diagnosed using the criteria ÷ total number of live births) × 1000]; (ii) severe maternal outcome ratio per 1000 live births, calculated as: [(number of maternal deaths + number of maternal near misses) ÷ total number of live births × 1000]; (iii) ratio of maternal near miss to maternal death (number of cases of maternal near miss ÷ the number of maternal deaths); and (iv) mortality index calculated from: [number of maternal deaths ÷ (number of women with maternal near miss + number of maternal deaths) × 100]. The mortality index and the maternal near miss to mortality ratio indicate the quality of care; the lower the mortality index and the higher the maternal near-miss to maternal death ratio, the higher the quality of care.

### Ethical approval

The study was approved by the scientific advisory committee and following the standards set by the ethics committee (human studies) of Jawaharlal Institute of Postgraduate Medical Education and Research, Puducherry, India. Informed consent was obtained from all women enrolled in the primary study or their relatives. The ethics committee (human studies) approved the primary research proposal leading to the work submitted (vide no. JIP/ IEC/2013/3/173).

## Results

Among the 38 292 deliveries at the hospital from May 2018 to April 2021, there were 37 590 live births. Using the WHO severity indicators, we identified 1833 women with potentially life-threatening conditions (48.8 per 1000 live births). Among them, a total of 57 women died due to complications of birth (151.6 maternal deaths per 100 000 live births). The characteristics of the women at recruitment to the study and details of their pregnancy care are shown in Table 2. The most common condition among women with potentially life-threatening conditions was hypertension (1039 women; 56.7%), followed by other systemic or medical disorder. Categories of potentially life-threatening conditions and the case fatality rates of each condition in the study population are shown in Table 3.

**Table 2:** Sociodemographic characteristics among women with potentially life-threatening conditions and those who developed severe maternal outcomes, Puducherry, India, May 2018 to April 2021

Characteristic	Potentially life-threatening conditions (*n* = 1833)	Severe maternal outcomes (*n* = 380)
**Age, mean (SD) years**	26.6 (4.9)	27.4 (5.3)
**Marital status, no. (%) of women**		
Living with partner	1821 (99.3)	378 (99.5)
Single or divorced	12 (0.7)	2 (0.5)
**Parity, no. (%) of women**		
Nulliparous	1103 (60.2)	186 (48.9)
Primiparous	485 (26.5)	119 (31.3)
Multiparous	245 (13.4)	75 (19.7)
**Socioeconomic status, no. (%) of women^a^**		
Upper high	104 (5.7)	21 (5.5)
High	406 (22.1)	65 (17.1)
Upper middle	584 (31.9)	124 (32.6)
Lower middle	388 (21.2)	91 (23.9)
Poor	351 (19.1)	79 (20.8)
**No antenatal care visits, no. (%) of women**	26 (1.4)	9 (2.4)
**Timing of the maternal near-miss events, no. (%) of women**		
Antenatal	1678 (91.5)	309 (81.3)
First trimester (1–12 weeks)	52 (3.1)	11 (3.6)
Second trimester (13–28 weeks)	164 (9.8)	51 (16.5)
Third trimester (> 28 weeks)	1462 (87.1)	247 (79.5)
Postpartum	139 (7.6)	60 (15.8)
Post-abortion	16 (0.9)	11 (2.9)
**Timing of delivery, no. (%) of women^b^**		
Extreme pre-term (28–34 weeks)	341 (18.6)	87 (22.9)
Pre-term (34–37 weeks)	443 (24.2)	95 (25.0)
Term (≥ 37 weeks)	790 (43.1)	99 (26.1)
**Mode of delivery, no. (%) of women (*n* = 1574)^b^**		
Vaginal delivery	740 (47.0)	94 (33.5)
Caesarean section	834 (53.0)	187 (66.5)

^a^ We used the classification of Dalvi et al., 2020^24^ for socioeconomic status based on the per capita monthly income in Indian rupees (INR): upper high (7533 INR and above); high (3766–7532 INR); upper middle (2260–3765 INR); lower middle (1130–2259 INR); poor (below 1130 INR).

^b^ After excluding 259 women who had abortion and ectopic pregnancy among those with a potentially life-threatening condition, and 99 among those with severe maternal outcome.

Note: *n* is the total number of women surveyed. Potentially life-threatening conditions and severe maternal outcome were defined by the World Health Organization severity indicators.^2^

**Table 3:** Categories of potentially life-threatening conditions and the case fatality rates of each condition in the study population, Puducherry, India, May 2018 to April 2021

Condition	No. (%) of women			Case fatality rate, %
	Potentially life-threatening conditions (*n* = 1833)	Maternal near miss (*n* = 323)	Maternal death (*n* = 57)	
**Haemorrhage disorder**	206 (11.2)	96 (29.7)	19 (33.3)	9.2
Post-abortion haemorrhage	27 (1.5)	11 (3.4)	4 (7.0)	14.8
Ectopic pregnancy	21 (1.1)	6 (1.9)	0 (0.0)	0.0
Gestational trophoblastic disease	5 (0.3)	4 (1.2)	0 (0.0)	0.0
Placental abruption	60 (3.3)	30 (9.3)	4 (7.0)	6.6
Ruptured uterus	16 (0.9)	4 (1.2)	2 (3.5)	12.5
Morbidly adherent placenta	29 (1.6)	18 (5.6)	0 (0.0)	0.0
Postpartum haemorrhage	90 (4.9)	45 (13.9)	12 (21.0)	13.3
**Infection, timing**	100 (5.5)	37 (11.5)	8 (14.0)	8.0
Antenatal	58 (3.2)	19 (5.9)	5 (8.8)	8.6
Intrapartum	5 (0.3)	1 (0.3)	0 (0.0)	0.0
Postpartum	34 (1.9)	17 (5.3)	2 (3.5)	5.8
Post-abortion	3 (0.2)	0 (0.0)	1 (1.8)	33.3
**Hypertensive disorder**	1039 (56.7)	155 (48.0)	16 (28.1)	1.5
Gestational hypertension	489 (26.7)	64 (19.8)	6 (10.5)	1.2
Pre-eclampsia	656 (35.8)	93 (28.8)	13 (22.8)	1.9
Eclampsia	61 (3.3)	20 (6.2)	1 (1.8)	1.6
Hypertensive encephalopathy	14 (0.8)	0 (0.0)	0 (0.0)	0.0
HELLP syndrome	104 (5.7)	56 (17.3)	5 (8.8)	1.9
**Medical disorder**	1181 (64.4)	227 (70.3)	38 (66.7)	3.2
Anaemia	438 (23.9)	120 (37.2)	17 (29.8)	3.9
Endometritis	10 (0.5)	0 (0.0)	0 (0.0)	0.0
Thyroid crisis	295 (16.1)	48 (14.9)	2 (3.5)	0.7
Seizures	92 (5.0)	20 (6.2)	7 (12.3)	7.6
Heart disease	281 (15.3)	38 (11.8)	13 (22.8)	4.6
Pulmonary oedema or respiratory failure	82 (4.5)	33 (10.2)	12 (21.1)	14.6
Diabetes	316 (17.2)	42 (13.0)	6 (10.5)	1.9
**Labour-related disorder**	17 (0.9)	3 (0.9)	0 (0.0)	0.0
Prolonged or obstructed labour	14 (0.8)	2 (0.6)	0 (0.0)	0.0

HELLP: haemolysis, elevated liver enzymes and low platelet count.

Note: n is the total number of women surveyed.

According to the WHO criteria, 380 of the women had severe maternal outcomes. Among these women, receiving blood transfusion of more than five units was the most common criterion met out of the WHO near-miss criteria set (108 women, 28.4%; Table 4). 

**Table 4:** WHO maternal near-miss indicators among women with severe maternal outcomes, Puducherry, India, May 2018 to April 2021

Criterion	No. (%) (*n* = 380)
**Clinical**	
Overall	184 (48.4)
Acute cyanosis	0.0 (0.0)
Loss of consciousness lasting > 12 hours	14 (3.7)
Gasping	1 (0.3)
Cardiac arrest	3 (0.8)
Respiratory rate > 40 or < 6 per minute	18 (4.7)
Stroke	2 (0.5)
Shock	31 (8.2)
Uncontrollable fit	19 (5.0)
Total paralysis	3 (0.8)
Oliguria, non-responsive to fluids or diuretics	25 (6.6)
Jaundice in the presence of pre-eclampsia	6 (1.6)
Failure to form clots	93 (24.5)
**Laboratory**	
Overall	193 (50.8)
Oxygen saturation < 90% for > 60 minutes	48 (12.6)
pH < 7.1	14 (3.7)
PaO_2_/FiO_2_ < 200 mmHg	4 (1.1)
Lactate > 5 mmol/L or > 45.0 mg/dL	4 (1.1)
Creatinine > 300 mmol/L or > 3.5 mg/dL	24 (6.3)
Acute severe thrombocytopenia (< 50 000 platelets)	102 (26.8)
Bilirubin > 100 mmol/L or > 6.0 mg/dL	26 (6.8)
**Management based**	
Overall	268 (70.5)
Use of continuous vasoactive drugs	18 (4.7)
Intubation and ventilation for > 60 minutes not related to anaesthesia	80 (21.1)
Hysterectomy following infection or haemorrhage	35 (9.2)
Dialysis for acute renal failure	19 (5.0)
Transfusion of > 5 units red cell transfusion	108 (28.4)
Cardiopulmonary resuscitation	30 (7.9)

WHO: World Health Organization.

Note: Women were identified using the criteria proposed by WHO for diagnosis of obstetric near miss.^9^ Women could have one or more criteria identified.

Applying the different sets of criteria to the data, we found that the numbers of women classified with severe maternal outcome ranged from 333 to 641 and the number of near misses identified ranged from 280 using the proposed Indian national criteria to 588 using the CDC criteria (Table 5). The corresponding incidence of near miss in the study sample therefore ranged from 7.6 to 15.6 per 1000 live births. These variations also affected the indices of quality of care (mortality index and maternal near-miss to mortality ratio) in the study population. 

**Table 5:** Comparison of different criteria sets for identifying maternal near miss and severe maternal morbidity, Puducherry, India, May 2018 to April 2021

Indicator	Mantel et al., 1998^18^	Waterstone et al., 2001^19^	Roberts et al., 2008^7^	WHO criteria, 2011^9^	Indian national criteria, 2014^23^	Chou et al., 2016 (Global Network criteria)^15^	CDC criteria, 2017^20^
Severe maternal outcome, no. of women	372	375	514	380	333	540	641
Maternal near miss, no. of women	324	354	462	323	280	483	588
Maternal deaths not counted,^a^ no. of women	9	36	5	0	4	0	4
Severe maternal outcome ratio, per 1000 live births	9.9	10.0	13.7	10.1	9.0	14.4	17.1
Maternal near-miss ratio, per 1000 live births	8.6	9.4	12.2	8.6	7.6	12.8	15.6
Mortality index, %	12.9	5.6	10.1	15.0	15.7	10.6	8.2
Maternal near-miss to maternal death ratio	6.75:1	16.8:1	8.8:1	5.7:1	5.35:1	8.47:1	11:1

CDC: United States Centers for Disease Control and Prevention; WHO: World Health Organization.

^a^ Actual maternal deaths among the study sample were 57.

Note: We applied each set of criteria to data from 1833 women who had potentially life-threatening conditions as defined using the WHO severity indicators.^2^ Total live births in the hospital over the time period were 37 590.

The WHO criteria identified 323 women with near miss and all 57 women who died. Use of ICD-based diagnosis in two criteria sets^7^^,^^20^ increased the number of women classified with near miss (to 462 and 588 women, respectively), but few women who died from the complications were overlooked (only 5 and 4 women, respectively). However, the Waterstone criteria^19^ and Mantel criteria^18^ missed more maternal deaths (36 and 9 women, respectively). Using the Indian criteria, only 280 women were classified as near miss, and four women who ultimately died due to complications were not identified. 

**Fig. 1:**
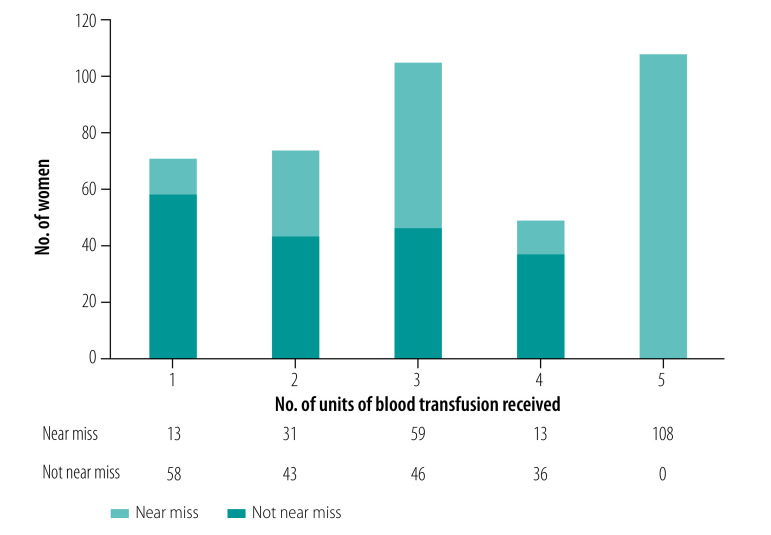
Number of women diagnosed with near miss according to the WHO criteria when categorized based on the number of blood transfusions received, Puducherry, India, May 2018 to April 2021

Use of any number of blood transfusions in three of the criteria sets^7^^,^^15^^,^^22^ also increased the total number of women diagnosed with maternal near miss. Only the WHO^2^ and Global Network criteria^15^ identified all 57 women who died due to the various conditions (Table 5). Fig. 1 shows the incidence of near miss according to the number of units of transfusion the women received according to WHO criteria. There were 407 women who received one or more units of transfusion who were identified using the criteria where any volume of blood was transfused. These results also show that all the women receiving five or more units of blood were identified as maternal near misses, whereas if a cut-off of four or fewer units were used many would be counted as near miss despite not having a life-threatening (near miss) event. 

WHO: World Health Organization.

Note: We applied each set of criteria to data from 1833 women who had potentially life-threatening conditions as defined using the WHO severity indicators.^2^ Total live births in the hospital over the time period were 37 590.

## Discussion

Potentially life-threatening complications occurred in 48.8 per 1000 live births in the study period and there were 57 maternal deaths. Among the 323 women identified as near miss using the WHO tool, two thirds of the women fulfilled one or more of the management criteria. Among the seven criteria sets used in various settings worldwide for identifying maternal near miss, the WHO and the Global Network criteria identified women with severe maternal outcomes without missing those who died due to the events. Use of any number of blood transfusions as an indicator increased the total number of women diagnosed with maternal near miss.

To date, maternal mortality is considered an important indicator of health care and is used to compare health-care systems and the gaps in various settings across the globe.^25^ Analysis of maternal near miss or severe maternal morbidity can provide more information about the standard of care and help assess the quality of health-care systems.^2^ Even after the introduction of the near-miss tool by WHO in 2011, a literature search indicates the wide variation in the use of criteria for identifying severe maternal morbidity across health systems between and within countries.^4^^–^^6^^,^^10^^–^^14^^,^^22^ Lack of availability of the investigations, facilities or expertise to adopt the WHO criteria is often cited as a reason for using modifications or other criteria, especially in low- or middle-income countries.^12^^,^^22^^,^^26^

Even though different sets of criteria are available for identifying cases, there is considerable overlap in the individual indicators defined.^18^^,^^19^^,^^22^^,^^26^ The Mantel and Waterstone criteria use organ dysfunction or a specific condition-based approach to diagnose near miss.^18^^,^^19^ Since many of the severity indicators in the WHO criteria are not used in these criteria sets, the ability of these classifications to detect near misses is limited, resulting in an underestimate of the women who died after suffering a life-threatening condition, as seen in the present analysis.

In high-income countries, where electronic health records or population databases are available, ICD-based criteria sets easily identify women with near-miss births.^7^^,^^20^^,^^22^ Although accessibility of the records and the standardization of the data can be an advantage in such criteria sets, there can still be issues with diagnostic criteria sets that incorporate laboratory criteria and rely primarily on the condition and the management received by the woman. We found that the criteria set that used ICD-based criteria,^7^^,^^20^^,^^22^ for example, led to underestimating the number of women with severe complications or who died.

In an attempt to follow the WHO mandate to initiate near-miss reviews in all settings in all countries, India’s national technical group in 2014 proposed a set of local-specific expert consensus criteria.^2^^,^^16^ The indicators were proposed under four sections: (i) disorders and conditions or complications; (ii) clinical findings (symptoms and signs); (iii) results of investigations; and (iv) interventions, with each condition or complication further having a subsection which includes (i) clinical feature or (ii) investigation and (iii) management or intervention received, to identify the condition. To identify a woman with near miss, the method requires a minimum of three criteria to be met in each section – one each from clinical findings (either symptoms or signs), investigations carried out, and interventions carried out to manage the care – or any single criterion which signifies cardiorespiratory collapse.^16^^,^^23^ The subcriteria resulted in a system with more than a hundred possible indicator variables, and the minimum criteria (that is, requirement of one each of the three criteria) to diagnose a near miss resulted in underestimating near-miss events, including women who died due to complications.

Recognizing the limitations of the WHO criteria in a low-resource setting where the investigations and facilities are limited and there are high proportions of community or home deliveries, the Global Network criteria were proposed in 2016.^15^^,^^26^^,^^27^ The criteria set is similar to the WHO criteria but omitting the indicator of laboratory test results while adding the change to any number of blood and blood products transfusion. As evident from our study, laboratory criteria are indirect markers of the indicators under the WHO management and clinical criteria. The Global Network criteria also identified all women with severe morbidity, including those who died due to the complications.

In contrast with the WHO criteria, three other criteria sets use the transfusion of any volume of blood products, irrespective of the number of units received, to identify a pregnant woman with severe maternal morbidity.^7^^,^^15^^,^^20^ Even when the Global Network criteria identified all those who died from complications, the criterion of transfusion of any blood products without specifying the number of units exaggerates the number of women diagnosed with near miss (similar to the ICD-based systems).^7^^,^^22^ This finding may limit the usefulness of the Global Network approach in low- and middle-income countries. Many women who have moderate to severe anaemia receive a blood transfusion during pregnancy and childbirth to avert the complications, rather than being transfused following a life-threatening event such as obstetric haemorrhage, as is more often the case in high-income settings.

There were strengths and limitations to our study. Most of the earlier reports compared one or two systems of identifying near miss and used the WHO criteria as the gold standard.^4^^–^^6^^,^^11^^,^^13^ Meticulous screening using the condition or diagnosis given as per the ICD in two of the criteria sets^7^^,^^20^ can be considered a strength of this study. We compared seven methods, without assigning one as a reference standard for near miss, and presenting the results as frequencies and as the rates for various maternal near-miss indicators. This approach helps to reflect the actual burden on the health systems, which may be increased, and to identify women who may be missed yet died from complications. This study from a lower-middle-income country also highlights the need for a unified system applicable across various settings. As the hospital is considered as a regional referral centre for high-risk pregnancies and postnatal complications in the south-eastern region of India, the data might reflect the actual rates of near miss in the area and can also add to the strength of the study. Although the study had a large sample size, the investigation was conducted in a single tertiary hospital and this may limit the generalizability of the results; further evaluation would be needed in multicentre studies.

Since maternal mortality ratios are declining globally, validation of the criteria in various settings is required to enable comparisons and to identify areas for improvement.^25^ A set of uniform criteria, with minimum indicators to identify all women with severe maternal morbidity, validated across various settings, needs to be explored. As with the maternal morbidity ratio, near-miss rates could then be used as a health indicator. Such an approach also needs to consider differences in the presentation and the disease condition, as wide variations were observed between high-income and low-income settings in the causes of maternal mortality.^25^ As we observed, the use of either WHO or the Global Network criteria, especially in low- and middle-income countries, can be beneficial for identifying maternal near miss across various settings, which will allow the comparison of the quality of care across regions.

In conclusion, a uniform validated set of criteria that will aid in identifying near-miss births and allow comparison of health systems across the globe is needed. The WHO and Global Network tools may serve that purpose, as they aid in identifying all women with maternal near miss including those who died from maternal complications, even in lower-resource settings. The usefulness of blood transfusion as an indicator for near-miss events, without specifying the number of units, needs to be evaluated in future, especially in low- to middle-income countries where the criterion may not identify severe maternal morbidity.
